# Explaining stress coping behaviors in patients with multiple sclerosis based on the PRECEDE model: a qualitative directed content analysis

**DOI:** 10.1186/s12888-021-03643-y

**Published:** 2021-12-20

**Authors:** Atefeh Homayuni, Zahra Hosseini, Sedigheh Abedini

**Affiliations:** 1 Student Research Committee, Hormozgan University of Medical Sciences, Bandar Abbas, Iran; 2 Health Promotion and Education, Tobacco and Health Research Center, Hormozgan University of Medical Sciences, Bandar Abbas, Iran; 3 Health Promotion and Education, Social Determinants in Health Promotion Research Center, Hormozgan Health Institute, Hormozgan University of Medical Sciences, Bandar Abbas, Iran

**Keywords:** Coping behaviors, Directed content analysis, Multiple sclerosis, PRECEDE model, Stress

## Abstract

**Background:**

Stress can be regarded as one of the consequences of Multiple Sclerosis (MS) and a factor in exacerbating or recurring the symptoms resulting from the disease. This study aimed to explain the stress coping behaviors in patients with MS based on the PRECEDE model.

**Methods:**

This study is a qualitative directed content analysis research based on the PRECEDE model. Data were obtained through in-depth semi-structured interviews with 26 patients with MS, who were selected using a purposive sampling and maximum diversity in terms of gender, age, education, marital status, and employment. Data collection continued until the saturation occurred. Simultaneously, collected data were analyzed using a qualitative directed content analysis method.

**Results:**

Data analysis led to the identification of 11 sub-categories. Of these, 10 sub-categories were assigned to three categories of predisposing factors (awareness, attitude, self-efficacy, and perceived severity), enabling factors (existence of resources, access to resources, skills of using resources, and educational preferences), and reinforcing factors (social support, important others and behavioral consequences). The social comparison category was a new category identified from the analysis of interviews.

**Conclusions:**

Based on the results, individual, environmental and social factors play a role in the stress of these patients. Designing programs that lead to their empowerment and improvements in the environmental and social conditions can be effective in controlling stress in these patients. Based on the results, planners can adopt the most appropriate strategies to change these determinants, help reduce stress, and promote the psychological standard of living in these patients.

## Introduction

Multiple Sclerosis (MS) is a multifactorial, central nervous system, immune-mediated disease characterized by demyelination, neurodegeneration, inflammation, and gliosis [1], with an age of onset between 20 and 40 years [2]. Estimates show that there are approximately 913,925 adults with MS living in the United States and 2.3 million people living with the disease worldwide.

This disease leads to the emergence of a wide range of physical, emotional, and cognitive symptoms. Common physical symptoms include fatigue, sleep disorder, numbness or stinging, weakness, dizziness, sexual problems, pain, itching, movement problems, vision problems, and bladder, and bowel problems, which vary among adults. Mood swings, irritability, depression, and reduction of concentration ability, process incoming information, learn and memorize new information, organize, and problem solve are among the common emotional and cognitive disorders [3].

Today, psychological tension or stress is proposed as an aggravating factor and less likely as a MS- causing factor. In 85–90% of cases of MS, there are courses of exacerbation of symptoms and recovery, but the process of the disease is unpredictable and it is known today that psychological tensions cause the activation of this disease [4]. A longitudinal study by Ackerman et al. [5] on 23 women with MS which followed for 1 year, specified that 85% of MS exacerbations were associated with stressful life events in the preceding 6 weeks and stress was proposed to be an activating factor in the disease recurrence. Recent reports from the American Neurological Association also show that one of the most important exacerbating factors of MS is the stress resulting from life events. Researchers of this university investigated 73 MS patients and found that there was a significant relationship between patients’ reports of stressful life events and the exacerbation of their disease. There is a significant increase in risk of exacerbation in multiple sclerosis after stressful life events [6]. The findings of Gold et al. [7], which aimed to determine the role of stress-response systems for the pathogenesis and progression of MS, showed that the immune function by the major stress systems is impaired in MS. According to these researches, therefore, there is probably a strong relationship between stress and the exacerbation of MS, and it seems that reducing and controlling stress is very important in this disease.

As MS patients have to deal with both the stresses of daily life and those resulting from fluctuating and unpredictable disease symptoms, the progression of the disease may interfere with work, family life, communication, and social activities [8]. The purpose of stress management is to help these people cope with the mentioned challenges, which increases people’s ability to reduce stress and adapt appropriately to stressful situations. This intervention consists of elements, such as increasing awareness about stress, problem-solving training, assertiveness skills training, anger management, self-management, activities planning, and so on [9].

There are numerous theories of health behavior in the scientific literature, each of which attempts to explain why people engage in a behavior or fail to engage in that behavior [10]. In this study, a conceptual and scientific framework, namely the PRECEDE model, was used to identify the factors associated with stress coping behaviors. This model is one of the most famous and common planning models in the field of health education and health promotion, developed by Green and Kreuter [11]. According to Green and Kreuter [11], behavioral factors, as determinants of a particular behavior, can be classified as predisposing, enabling, and reinforcing factors. Predisposing factors are predictors of behavior that include knowledge, attitudes, beliefs, values, perceptions, existing skills, and self-efficacy. Enabling factors are predictors of behavioral and environmental changes, including accessibility, availability, rules, and policies. Reinforcing factors are factors that follow a behavior and provide ongoing rewards or motivations and include social support and important others [12]. The usefulness of educational planning based on the PRECEDE model has been confirmed in the reduction of psychological problems, such as reductions in anxiety [13], depression [14], and stress [15–17].

MS is highly prevalent in our country and especially among the young generation and women, particularly in Isfahan. This necessitates the ever-increasing need for appropriate interventions to reduce stress levels in patients with MS given the role of stress in the exacerbation of the disease, psychological and physical problems resulting from the disease, no definitive treatment, and failure of medical therapies in its effective treatment. Therefore, this study aims to explain stress coping behaviors in patients with MS based on a qualitative research approach. Therefore, the research question was proposed:

1. Which factors influence on the adoption of stress coping behaviors in patients with MS based on the PRECEDE model?

The obtained results can help us in designing and implementing effective intervention programs to reduce stress in these patients through the use of stress coping behaviors relying on predisposing, enabling, and reinforcing factors.

## Methods

### Study design

This study is based on a qualitative directed content analysis. in this approach, the initial coding starts from a theory, and the selected theory can help to focus on the research question. On the other hand, theory can help to predict interesting variables or relationships between variables. This method was presented by Hsieh and Shannon in 2005 [18]. The PRECEDE model was used in this research.

### Study setting and participants

Based on purposive sampling with maximum diversity (in terms of gender, age, education, marital status, occupation and type of MS), 26 patients were selected among those referred to the Isfahan MS Association. In purposeful sampling, information-rich cases related to the phenomenon are identified and selected. Persons who can provide the needed information to address research questions. The inclusion criteria of individuals with MS were: 1) having MS diagnosed by a neurologist, 2) having MS for more than 1 year, 3) Having not a chronic disease other than multiple sclerosis, and 4) being able to participate in the interview and sharing their experiences. Individuals were excluded if patients were unable to cooperate and talk due to the worsening of the disease or other reasons and were not willing to continue the interview at the time of the interview.

Firstly, the required permissions were obtained. According to the previous coordination with the head of research affairs of the MS Association, the researcher attended the classes of the association and after establishing initial communication with patients, introduced herself and explained about the purpose and importance of the study as well as the conditions for inclusion in the study. The researcher also posted an online advertisement on the MS Association website and installed an printed advertisement in the MS Association. In order to participate in the study, a number of patients voluntarily announced their readiness. Finally, the necessary arrangements were made to interview those who were willing to participate in the study.

### Data collection

Data were collected through semi-structured and in-depth interviews. Date collection continued until the saturation of data (a point in the data collection where no new categories emerge). Saturation was reached with 22 patients but we continued interview with 4 additional patients in order to provide greater confidence in the reliability of the study findings. The duration of each interview varied from 30 to 90 min depending on the participants’ interest and tolerance. The location of the interviews was chosen by the participants, which was at the patient’s living place or in a dedicated room in the MS Association. At the beginning of each session, the interviewer introduced herself and explained about the interview and the objectives of the present research. The interviews were recorded by a voice recorder.

The interview process began with questions about the participants’ demographic information, including their age, age of disease onset, level of education, marital status, and so on. The interview questions were based on the constructs of the educational factors of PRECEDE model, which began and continued with the following questions:

1. What do you know about stress?
2. Do you know about the consequences of stress?
3. Tell me about your skills and abilities to cope with stress.
4. In your opinion, what skills should you learn to control stress?
5. Tell me about your experiences regarding environmental barriers to coping with stress.
6. Tell me about your experiences regarding the role of family members, health care providers, friends, and others in performing stress coping behaviors.
7. Tell me about your feelings after doing stress coping behaviors continuously.
8. What problems did you have, when performing stress coping behaviors?

Besides, in-depth and exploratory questions were asked during the interviews and in accordance with the answers to elaborate on the details. These questions include: “Please explain more about it”, “Can you provide an example so that I can better realize your perception of ....?”, “Please let me know If you have a memory in this regard”, “When you say ...., what do you mean?”, “What feeling did you have in this regard?”, “Why and how?”

### Data analysis

Interviews were first handwritten on the paper and then typed as soon as possible. The text was read several times to gain a deep understanding of it and was broken down into the smallest meaningful units (codes). Based on the research question in the directed content analysis, coding can begin with one of these two ways: 1. If the purpose of the research is to identify and categorize all cases related to a particular phenomenon, the entire text should be read and those sections should be marked that are specified based on the researcher’s initial impression. In the next stage, the marked sections are coded based on predetermined codes (according to the theory). A new code can be given to each section of the text that does not fit into this initial coding. 2. Instant coding using predetermined codes, we identified data that cannot be encoded and then analyzed to determine if they are a category or a subcategory of existing codes. In this study, the first way was selected for encoding. To facilitate the organization and analysis of the qualitative data, the Max-QDA version 10 software was used.

### Ethical considerations

The present research was approved by the Ethics Committee of Hormozgan University of medical sciences (IR.HUMS.REC.1399.065). To ensure voluntary participation in the study, participants were asked to give their consent. All participants were given consent forms to sign. To observe the ethics in the research, the participants were explained about recording their voices while doing the interview. It was also emphasized that all received information was confidential and used only to achieve the objectives of the study. The interviewees were free to withdraw from the interview upon their request.

### Consideration of rigor

Four criteria of dependability, credibility, confirmability, and transferability were used to determine the accuracy of the data in this study. It was also tried to increase the credibility of the finding by devoting sufficient time for data collection, reviewing the extracted manuscripts and codes by participants and sending data to colleagues and using their supplementary comments. To increase the transferability of findings, a complete description was presented about the characteristics of the participants and all research processes, along with examples of the participants’ statements, to make it possible for other researchers to follow the research path.

## Results

### The participants’ characteristics

Participants ranged in age from 25 to 49 years with a mean age of 36.11 years. Married patients participating in the study had a minimum of 1 and a maximum of 3 children. Five participants had a history of MS in their relatives. All patients received health insurance services. The majority of the participants were in Relapsing-Remitting stage of MS.

Other demographic information of the participants is summarized in Table 1.

**Table 1:** Demographic characteristics of participants

Variable	N (percentage)
Marital status	
Single	7 (26.92)
Married	14 (53.85)
Widow	2 (7.69)
Divorced	3 (11.54)
Education level	
Junior high school	1 (3.85)
High school and diploma	13 (50)
Associate degree	3 (11.54)
Bachelor’s degree and higher	9 (34.61)
Occupation status	
Housewife	22 (84.61)
Employed	3 (11.54)
Retired	1 (3.85)
Frequency of hospitalization in the last 1 year	
None	17 (65.38)
Once	4 (15.39)
Twice or more	5 (19.23)
Frequency of the recurrence of disease within the last 1 year	
None	17 (65.39)
Once	6 (23.07)
Twice	2 (7.69)
More than twice	1 (3.85)

Eventually, data analysis revealed 11 sub-categories, which were divided into three predetermined categories and one newly identified category. These categories are predisposing factors, enabling factors, reinforcing factors, and social comparison. The categories and sub-categories are described in the following.

#### Predisposing factors

Predisposing factors indicate that adopting or changing a behavior requires a number of factors that precede the change of behavior and provide motivation to perform a behavior and cause the logic of that behavior. These factors in our study include four factors (sub-categories):

##### Awareness

This factor indicates the level of knowledge and information of patients about stress and the coping styles. Some participants did not have the necessary awareness about stress coping styles and this led to the adoption of inappropriate and inefficient coping styles, such as taking sedatives.

“Sometimes when I get stressed I do not know what to do? I do not have any experience. Unless my stress is finished over time” (40-year-old patient).

“I do not know what really help reduce my stress. Sometimes people resort to pills” (27-year-old patient).

Most of the participants were also interested in gaining knowledge about the concept of stress and its origin, and strategies for stress control and management. Some also wanted to increase MS disease-centered radio and television programs, because they believed that increasing the awareness of family members and the society about this disease would reduce the stresses imposed by others and increase support of the patient at the time of stress.

“I would like to know more about stress is and the controlling ways, and learn to cope with and accept stress and the problem-solving methods” (29-year-old patient).

“I would like to know how to control stress when I am stressed or to do something that the stress does not have much effect on me mentally and physically” (39-year-old patient).

“Not everyone is interested in reading books, but most of them watch television. I wish the radio and television had much more maneuver on this disease, its introduction, and the role and duties of companions in relation to a patient” (40-year-old patient).

##### Attitude

The participants had different positive and negative perspectives about stress, stress consequences, sources of stress, and stress coping styles. Most of them believed that stress caused the recurrence of MS and had negative effects on their physical health. Participants also expressed a wide range of beliefs related to the cause of stress, including the effects of fear, the environment and companions, obsession, indoctrination, and so on.

“Stress is the cause of all diseases, from the smallest to the largest disease. It is a trigger to start all diseases” (33-year-old patient).

“If you want to interpret stress in view of my disease, stress is a poison for us” (31-year-old patient)

Some participants also had completely different views about stress coping styles, such as attending counseling sessions, medication, thought management, and so on.

“If they want to hold a class to help us cope with stress, it would not be useful” (31-year-old patient).

“I concluded that the drugs we take, control the stress one hundred percent. Because I got a lot better than the beginning. My husband says you look to be a little calmer than before” (36-year-old patient).

##### Self-efficacy

Some of the participants believed in their ability to prevent stressful situations, control stress, perform stress coping behaviors, and their ability to teach stress coping behaviors to their companions if they learned these behaviors.

“I try to change my thoughts temporarily and do something else. I usually do not let stress stay with me, I try to help myself a lot” (29-year-old patient).

However, most participants felt that they were not able to cope with stress, or that they had inefficient ability in doing stress coping behaviors. The disabilities expressed by participants included the inability to avoid stress, the limited ability to cope with stress, temporary effects of stress-controlling behaviors, and forgetting stress coping behaviors at the time of stress.

“Nothing can calm me down at that moment unless the passage of time. I will calm down over time” (34-year-old patient).

##### Perceived severity

Most patients referred to the detrimental consequences of stress and believed that stress exacerbated MS and its symptoms, such as impaired movement and imbalance, speech disorder, impaired bladder (frequent urination) and bowel functions, tingling, and trembling of organs.

“Stress may not affect the physical condition of others, but it immediately impacts the physical situation of those who have MS, and these effects are not sayable” (27-year-old patient).

“Stress causes my disease to recur” (35-year-old patient).

“If I have stress, my left hand starts tingling soon. It means that I was so stressed that this happened to me” (35-year-old patient).

#### Enabling factors

Enabling factors indicate that a number of preconditions are required to adopt or change a behavior. These preconditions are considered a behavioral or environmental change that creates motivation before doing that behavior and affects one’s behavior directly or indirectly through environmental factors. In the present study, these factors were obtained in the form of four subcategories as follows:

##### Existence of resources

Most patients mentioned the existence of resources and services, such as holding training sessions by specialist physicians, holding recreational camps and sports and counseling sessions by the MS Association and charity associations for special diseases, and providing books and training resources by specialist physicians and the MS Association.

“When I filed the case, my doctor gave me a book about MS that explained items such as physical, mental, and medicine problems. Then, they held group counseling sessions for us with a counselor holding a Ph.D. in psychology. In these sessions, we are 10-12 people, the counselor talks, we also have questions and answers, and transfer our information to each other; that is very good” (36-year-old patient).

On the other hand, some participants expressed discomfort and dissatisfaction with obstacles and problems, such as using instructors without academic education in the MS Association, disturbance, and disruption in the work of sports coaches by some officials, not holding appropriate training classes, and delay in repairing equipment and welfare facilities of the association. Finally, some of them believed that there was a need for creating and developing necessary and essential resources, services, and facilities. Furthermore, increasing training classes, welfare facilities, and individual counseling, providing a relaxing environment and space by family members for the patient, providing free services to MS patients, reducing the expenses of life, and increasing radio and television programs on the subject of MS were other issues stated in this field.

“Special patients, such as MS, should be provided with conditions that can freely use some services such as counseling. The patient should be able to use counseling services of the association free of charge, and to talk with counselor conveniently to be discharged and get guidance” (35-year-old patient).

##### Access to resources

Most of patients mentioned limitations that prevented them from access to resources and performing stress coping behaviors. Patients’ physical problems, traffic problems for going to the MS Association, lack of time, parental role responsibilities, and job responsibilities were among the personal and environmental deterrent factors, which they believed to create restrictions to perform stress coping behaviors.

“I used to do yoga when I was single. It was effective too. It helped me a lot to walk. After I got married and came from that neighborhood, I no longer had access to my instructor. Even when I had a child, it was very difficult for me to attended a yoga class” (38-year-old patient).

“At the beginning, I participated in MS Association seminars, but now that I am no longer living in Isfahan and I am far from there, I cannot take part in seminars” (48-year-old patient).

“I participated in TRX and Pilates classes and they were really effective. I am not exaggerating if I say that my stress dropped by 70-80%. But now I can no longer take part because of my back condition. This is the third time that I had a back operation” (40-year-old patient).

##### Skills of using resources

Most of the participants had little information about stress coping skills and felt that they did not have the necessary skills. They believed that familiarity with and acquisition of stress controlling and coping skills, such as communication and conversation skills, musical skills, and sensory and movement skills, could help them when facing stress. On the other hand, some of them believed that stress prevention was not possible, and no skill could help them to control and cope with stress.

“I like to learn communication and conversation skills” (29-year-old patient).

“I feel that stress occurs by itself in many places. Whatever you do, it goes its own way and does not require any skill” (37-year-old patient).

##### Educational preferences

Most of the participants wanted to hold training courses to control and cope with stress. They had various preferences in terms of the method of holding training (in-person or virtually), as well as the ratio of expertise, capabilities, and the required characteristics of a trainer.

“Certainly someone with education in psychology can help much better. That is a doctor that can help with physical activity and disease. For many years, I have had MS, and my doctor only controls my medicines and monitors my physical situation. When I talked to my doctor about my stresses, the answer I got was to take citalopram pills every morning” (25-year-old patient).

Participants also presented a variety of suggestions for holding these courses more effectively. Suggestions offered by the participants include: holding practical classes, holding training classes for families, and holding a training course with the presence of a spouse or a social friend.

“They should hold a series of classes for parents who are much stressed. Most of the time, I quarrel with them. I just laugh at their stress. My father would have stress about if I get married …” (31-year-old patient).

#### Reinforcing factors

Reinforcing factors state that for a behavior to continue, be repeated, and reinforced, it needs a number of factors to continuously provide a reward for maintaining the behavior and ultimately lead to the stability and continuity of that behavior. In the present study, these factors were obtained in the form of three subcategories:

##### Social support

Most of the participants mentioned the support and encouragement of family members (spouse, parents, sister, brother, etc.) and the community (sports coach, friends, university professors, etc.). These supports emerged in the form of emotional support and help in doing housework. Some patients noted that the emotional supports of family and companions and understanding their conditions had an effective role in increasing their self-confidence, improving their health status, and gaining peace of mind.

“Our TRX coach gave us wonderful self-confidence. I could run in front of her. I have never been able to run since 2006. I could run up and down the stairs without getting my hands on the railings. These were all her help” (34-year-old patient).

Some patients were also upset because their family members did not understand their conditions and support them.

“They say to me: Why are you stressed? Well, it does not have any stress. I feel they do not understand me. Maybe others really cannot understand me” (26-year-old patient).

##### Important others

Most participants felt that the recommendation and encouragement of family members (spouse, children, and parents), health personnel, relatives, friends, acquaintances, and other key people, such as sports coach and counselor, encouraged them to perform stress coping behaviors.

“I used to go to a yoga class for one year. Our instructor told me to perform relaxation when I was stressed or when I had insomnia. Take five deep breaths. Every night at bedtime, count the blessings that God has given to you, even write them down, and write down a new blessing every night. I did so and they were good to do” (47-year-old patient).

“I was thinking for a while about not going to a yoga class anymore. My mother quarreled with me and told me to go to the class. She told me to go and see my friends. Each of them has separate information. Each one has an experience. Go and use it. My mother strongly encouraged me to go to the class” (36-year-old patient).

##### Behavioral consequences

Patients who acquired a positive experience by doing stress coping behaviors were more encouraged to engage in such behaviors. According to the participants, these experiences were: lack of sleep problems, feeling calm, patience, happiness and mirth, and being understood by their peers.

“Yoga calms me down, I feel it, and this peace of mind relieves my stresses. Here, I can talk to the guys exactly about my stresses and everything in my heart that I cannot say these things at home. The guys understand me and are sick, as I am” (36-year-old patient).

In contrast, the experience of unpleasant feelings in a small number of patients prevented them to do such behaviors.

“I hate the yoga class because I talked to people who went to yoga class and I found out that I did not like sitting still in a place” (31-year-old patient).

#### Social comparison

Comparison of self with others, referred to as social comparison, is among the factors that can increase or decrease participants’ motivation to perform stress coping behaviors. Highly motivated patients were more likely to show desirable behavioral changes. This comparison was performed in participants in the form of a positive upward comparison (feeling hopeful that you can be as good as the person with whom you are comparing yourself), a negative downward comparison (feeling frustrated and worried that you may find yourself in a worse position than the person with whom you are comparing yourself), and a positive downward comparison (feeling good luck that you are not in a bad position as the person with whom you are comparing yourself).

“When I entered the MS Association for TRX classes, there were 2-3 guys, one of whom walked very badly; you know, there are a lot of people whose bodies are trembling. My mind tells me that my way will be like this in the future. I lose my spirit and try to take myself away from these groups” (34-year-old patient).

“I see in class that there are guys who are worse at walking than me. That’s why I say: Well, I am better than them. So, what is my problem?” (36-year-old patient).

## Discussion

The present study was aimed to identify the factors associated with stress coping behaviors in patients with MS. In this study, awareness, attitude toward stress, stress symptoms and coping strategies, self-efficacy, and perceived severity were proposed and identified as predisposing factors. In a study, Hosseini et al. [19] could increase the subjects’ attitude in a way that led to their increased tendency to acquire knowledge about stress coping methods. Kinchen and Loerzel [20] reported that undergraduate nursing students were open to using or recommendeing holistic therapies to relieve stress, but they considered the lack of knowledge and lack of time as barriers to their practice. Physical activity, prayer and meditation, time management, distraction, socialization, artwork, interaction with animals, and other activities were among the strategies used by students to manage stress from school or work. Apolinário-Hagen et al. [21] assessed determinant factors of public acceptance of stress management applications. They found that positive attitudes toward using mHealth to cope with stress, skepticism/perceived risks, and stress symptoms were among the most important predictors of accepting these applications.

In our study, some patients believed in their ability to prevent and cope with stress, a concept referred to as self-efficacy. For a behavior change or beginning of a new behavior to be successful, a person must be confident in their ability to overcome perceived obstacles and have a strong belief that a particular action will lead to a positive outcome [22]. Parschau et al. [23] presented evidence that the more people believe in their efficiency and ability to perform physical activity, the more they would perform the desired behavior.

The perceived severity indicates the point that individuals must understand the depth of the risk of stress and the seriousness of its various complications on their physical, psychological, social, and economic dimensions to adopt stress coping behaviors. Participants in this study mentioned the physical, emotional, cognitive, and behavioral effects of stress. In a study conducted by Lupien et al. [24], using data from human and animal studies, they examined the effects of stress during the prenatal period, infancy, childhood, adolescence, adulthood, and aging on the brain, behavior, and cognition. Some theorists believe that both hope and stress affect psychological well-being in difficult conditions. When the stressor affects a human’s life, their emotional state and physiological thinking deviate from normal and balanced level, the cognitive activity becomes vulnerable, and behavioral problems are recalled as the feelings of anxiety, depression, and stress [25].

Based on the results of the study, the subcategories of enabling factors include the existence of and access to resources, skills of using resources, and educational preferences. Enabling factors include those groups of skills, availability, and access to resources, regulations, rules, and barriers that can help or hinder behavioral or environmental changes [26]. Holding periodic training sessions by specialist physicians, providing books and training resources by specialist physicians and the MS Association, as well as holding recreational camps and sports classes and counseling by the MS Association and charity associations, were among the resources that increased patients’ ability to cope with stress. In this regard, Tudiver and Talbot [27] found out that the lack of access to doctors was one of the most important preventing barriers to seek help by men. In the study conducted by Sabzmakan et al. [28], patients and health workers also considered the existence of a psychologist to be necessary to provide counseling to diabetic patients for controlling stress.

It has been reported that the environment plays an important and changeable role, directly or indirectly through behavior in the cause of health problems [29]. The results of this study also confirm the indirect effects of the environment, which is environmental barriers to perform stress coping behaviors. Patients pointed to obstacles, such as using instructors without academic education, disturbance and disruption in the work of instructors by officials in MS Association, and delays in repairing the association’s equipment and facilities, as well as physical problems, traffic problems, lack of time and parental role responsibilities, and job responsibilities.

In this study, participants considered the lack of adequate skills in the field of stress management as one of the barriers to performing stress coping behaviors, and felt that learning skills, such as communication and conversation skills, movement and physical skills, and musical skills, could help them to be empowered. This finding is consistent with the results of studies conducted by Shauna et al. [30], Bridges et al. [31], and Didehvar et al. [32]. The results of study conducted by Didehvar et al. [32] on Iranian nurses and midwives showed that the mean score of learning stress management skills and the level of their adaption increased significantly in the two intervention groups after training. The findings of King et al. [33] also proved that health professionals play an important role in patient education, but they rarely receive training in effective teaching and counseling techniques. By training the patient effectively and problem solving, they improved several types of important skills in the control of blood sugar, and patients were able to overcome the self-care barriers of diabetes.

In this research, social support, important others, and behavioral consequences as reinforcing factors had an effective role in performing stress coping behaviors. Findings showed that social support is an important factor affecting patients’ quality of life and coping with stress. For most participants, this support received from family members and companions in the form of participation in doing housework, patient care, understanding the patient’s situation, and emotional support. The results of study conducted by Ozdemir et al. [34] on women with breast cancer showed that effective stress coping levels increased by increasing perceived support scores from family and total perceived support score. These researchers found that social support and age significantly predicted effective stress management. Yildirim et al. [35] also indicated that stress coping levels in nursing students were affected by self-esteem and social support. Social support is an important factor in maintaining human health and stabilizing health behaviors [36]. According to Bandura, after learning behavior, in order to a person’s learning to be translated into performance, there is a need for encouraging factors, including reinforcement, which can be provided by companions [37].

Another reinforcing factor in the present study for performing or not performing stress coping behaviors is the positive or negative experiences of the person after doing those behaviors. In the present study, patients who had a positive experience of performing these behaviors (such as resolving sleep problems, feeling calm, and patience) were more likely to be encouraged to perform the behaviors and felt good about doing these behaviors. This type of reinforcements is called internal or latent reinforcements, which refer to the feeling of satisfaction obtained from achieving a goal. These factors are the same as internal motivations, that is, situations in which when a person performs a behavior, enjoys, learns new things, and develops his/her inner abilities [38]. Stächele et al. [39] examined the effects of a 6-week internet-based cognitive-behavioral stress management (IBSM) program on perceived stress, coping skills, emotional exhaustion, depressive symptoms and sleep quality. They found that coping skills and sleep quality increased in the intervention group but the perceived stress reduced.

In our study, patients used different types of social comparisons, which encouraged them to perform stress coping behaviors. In a study conducted on chronic patients, Dibb and Yardley [40] concluded that participants compared themselves with others to gain information about their condition and how to cope with it. In their study, the worse health related quality of life was associated with seeking information and with making negative comparisons with people who were better off or worse off. Rogers et al. [41] also confirmed that the tendency to make positive comparisons result in beneficial self-evaluations. The positive comparison allows participants to show themselves as socially and morally worthy and valuable and makes them be present and activate in the community with self-confidence and more easily. Concerning people with chronic disease, it can be argued that the factor of hope is faded and the despair and sadness feelings will surround patients who think that their health condition will become the same as that of people whose disease is worse than theirs. As a result, such comparisons weaken a person’s mental status. In contrast, some patients think that their condition will be the same as that of those who have overcome their disease and regained their health. Such perceptions will make the window of hope in the hearts of such patients clearer, and as a result, while improving their mental status, will strengthen their efforts to achieve further improvements.

According to the findings of this study based on patients’ law familiarity with stress and its coping behaviors and since stress is a risk factor for MS and exacerbate it, it is suggested that health care professionals design and implement educational intervention programs aimed at preventing and reducing stress in MS Association and charity Associations. In addition, environmental barriers and restrictions in the MS Association should be removed and the support systems provided by these associations (such as increasing counseling classes, increasing sports and art classes and so on) should be increased. This study also included some limitations: First, all the participants were female because the men did not have the opportunity or patience to participate in the interview. The second was the use of the theory-centered directed qualitative content analysis that forces the researcher to act within a theoretical framework. However, interviewing with open-ended questions based on the PRECEDE model could explain the subcategories and categories of the PRECEDE model, and this is one of the advantages of the qualitative directed content analysis.

## Conclusion

One of the most important measures to prevent MS or control it in patients with MS is stress management. Findings obtained from the current study indicate that adopting maladaptive coping stress behaviors in patients with MS depends on various factors including: inadequate awareness or lack of it, inaccurate attitude, low self-efficacy, lack of the necessary skills to cope with stress, insufficient access to necessary resources, not receiving social support, and social comparison. Given that stress is a risk factor for MS disease, MS Association and charity associations can help to control and reduce stress in the patients by providing different services such as: informing patients about stress and adaptive coping styles, teaching stress management skills, holding family therapy courses, providing free counseling and psychology services, providing sports, art and educational services freely, providing the necessary training resources on stress and so on.

## Data Availability

The datasets used analyzed during the current study are available from the corresponding author on reasonable request.
